# Supplementary material for: Rapid turnover of life-cycle-related genes in the brown algae
Source: Genome Biol. 2019 Feb 14;20:35. doi: 10.1186/s13059-019-1630-6 (PMC6374913; doi:10.1186/s13059-019-1630-6)
Supplement: Supplementary file 3 — Protein alignments, corresponding Gblocks html file and CDS sequences for the lineage conserved gametophyte-biased genes. (ZIP 2109 kb) [file 13059_2019_1630_MOESM3_ESM.zip › Con_GA_alignments/SJ07936.aa_ali.fasta-gb.htm]

SJ07936.aa.fasta.trans.aa\_ali.fasta


## Gblocks 0.91b Results

Processed file: **SJ07936.aa.fasta.trans.aa\_ali.fasta**  
Number of sequences: **2**  
Alignment assumed to be: **Protein**  
New number of positions: **262** (selected positions are underlined in blue)

```
                         10        20        30        40        50        60
                 =========+=========+=========+=========+=========+=========+
SJ07936          MVAKRDRSPGVCLALLVAQLLGATLSSRNIEVTAAFYTNGNLPATSDTATEPASAWSNGK
Mac.72829.1      ------------------------------------------------------------
                                                                             


                         70        80        90       100       110       120
                 =========+=========+=========+=========+=========+=========+
SJ07936          AYKPKGRRLDAGEQRIAAERQQQHAVGRHLAATGQPLEARGGGELAAEGQRLSSAGGQRL
Mac.72829.1      ------------------------------------------------------------
                                                                             


                        130       140       150       160       170       180
                 =========+=========+=========+=========+=========+=========+
SJ07936          STSGGKRLSEARGPRLPAGGQQLSAAGGKQPAAGGQLLHAGGRQLAAEGQRLDAARGRRP
Mac.72829.1      ------------------------------------------------------------
                                                                             


                        190       200       210       220       230       240
                 =========+=========+=========+=========+=========+=========+
SJ07936          DTAGGRRQVDAAGSHQLGLDQENRNLNLLEEQPRRDGFRRDLLIANTKFFVDTCMEGQSF
Mac.72829.1      --------------QQLYQDVR-KTLNLKEE-PHRHASQRDLLIANTKFFVDTCMEGQSF
                                                 ############################


                        250       260       270       280       290       300
                 =========+=========+=========+=========+=========+=========+
SJ07936          TIASASHPELEGCYAADEVLGKVPPVTGQYVYSSARPVSAGGGKNVVATTSFFDPSFAED
Mac.72829.1      AVASENHPELEGCYCADAVLGKVPPVVGQYVYSSPRPASAGGGENVLATTSFVDPSFAED
                 ############################################################


                        310       320       330       340       350       360
                 =========+=========+=========+=========+=========+=========+
SJ07936          EYWVLMSTDSINARTKDNETVYCVSAQMANMFNHPSEVTWACDLTGKMEEIEEPSESDLT
Mac.72829.1      EYWVLMSTDSINGRSKDEETVYCVSAQMANMFNHPSEVTWACDLTGKMQEIKEPAASELT
                 ############################################################


                        370       380       390       400       410       420
                 =========+=========+=========+=========+=========+=========+
SJ07936          VACGCPGLPPLTRFDTEAIALRRQQMSAAGRRRKKNNNKNKNNRVYGANGGRSGSTSNSS
Mac.72829.1      VTCGCPGMPPLTRFDTEAADLRKERMSAAGRWRKKKNNSN-NKKTNGRNGG-----SSSS
                 ######################################## ##########         


                        430       440       450       460       470       480
                 =========+=========+=========+=========+=========+=========+
SJ07936          SRGSSSSFGVSLFHLFGLVCVFLAVVTRLRASRRRTQATAAAVKTVATRVSSGS-GGDAG
Mac.72829.1      STG--LSFGPSPFHLFGLICAFLAVVVRVRASRRRTHT--AKVIATASGVSGGSNSGGGG
                       ##############################    ##############      


                        490       500       510       520
                 =========+=========+=========+=========+======
SJ07936          KGGDAEEEAAVVGVGVGVSLLPSAAELTERRPSATATERTIPAQSV
Mac.72829.1      GGGEAGRSVDAAEAAVGISLLPSTVELTERRPSVTGR---------
                                ####################
```

```
Parameters used
Minimum Number Of Sequences For A Conserved Position: 2
Minimum Number Of Sequences For A Flanking Position: 2
Maximum Number Of Contiguous Nonconserved Positions: 8
Minimum Length Of A Block: 10
Allowed Gap Positions: None
Use Similarity Matrices: Yes
```

```
Flank positions of the 5 selected block(s)
Flanks: [213  400]  [402  411]  [427  456]  [461  474]  [496  515]  

New number of positions in SJ07936.aa.fasta.trans.aa_ali.fasta-gb:  262  (49% of the original 526 positions)
```
